# Overexpression of endothelin B receptor in glioblastoma: a prognostic marker and therapeutic target?

**DOI:** 10.1186/s12885-018-4012-7

**Published:** 2018-02-06

**Authors:** Suhas Vasaikar, Giorgos Tsipras, Natalia Landázuri, Helena Costa, Vanessa Wilhelmi, Patrick Scicluna, Huanhuan L. Cui, Abdul-Aleem Mohammad, Belghis Davoudi, Mingmei Shang, Sharan Ananthaseshan, Klas Strååt, Giuseppe Stragliotto, Afsar Rahbar, Kum Thong Wong, Jesper Tegner, Koon-Chu Yaiw, Cecilia Söderberg-Naucler

**Affiliations:** 1 Unit of Computational Medicine, Center for Molecular Medicine, Department of Medicine, Karolinska Institutet, Stockholm, Sweden; 2 Cell and Molecular Immunology, Experimental Cardiovascular Unit, Departments of Medicine and Neurology, Center for Molecular Medicine, Karolinska Institutet, SE-171 76 Stockholm, Sweden; 3 Department of Cell and Molecular Biology, Karolinska Institutet, Stockholm, Sweden; 4 Department of Neurosurgery, Karolinska University Hospital, Stockholm, Sweden; 5 Department of Pathology, University of Malaya, Kuala Lumpur, Malaysia; 6 Biological and Environmental Sciences and Engineering Division (BESE), Computer, Electrical and Mathematical Sciences and Engineering Division (CEMSE), King Abdullah University of Science and Technology (KAUST), Thuwal, 23955–6900 Kingdom of Saudi Arabia

**Keywords:** Glioblastoma, Endothelin B receptor, Endothelin receptor antagonists

## Abstract

**Background:**

Glioblastoma (GBM) is the most common malignant brain tumor with median survival of 12-15 months. Owing to uncertainty in clinical outcome, additional prognostic marker(s) apart from existing markers are needed. Since overexpression of endothelin B receptor (ETBR) has been demonstrated in gliomas, we aimed to test whether ETBR is a useful prognostic marker in GBM and examine if the clinically available endothelin receptor antagonists (ERA) could be useful in the disease treatment.

**Methods:**

Data from The Cancer Genome Atlas and the Gene Expression Omnibus database were analyzed to assess ETBR expression. For survival analysis, glioblastoma samples from 25 Swedish patients were immunostained for ETBR, and the findings were correlated with clinical history. The druggability of ETBR was assessed by protein-protein interaction network analysis. ERAs were analyzed for toxicity in in vitro assays with GBM and breast cancer cells.

**Results:**

By bioinformatics analysis, ETBR was found to be upregulated in glioblastoma patients, and its expression levels were correlated with reduced survival. ETBR interacts with key proteins involved in cancer pathogenesis, suggesting it as a druggable target. In vitro viability assays showed that ERAs may hold promise to treat glioblastoma and breast cancer.

**Conclusions:**

ETBR is overexpressed in glioblastoma and other cancers and may be a prognostic marker in glioblastoma. ERAs may be useful for treating cancer patients.

**Electronic supplementary material:**

The online version of this article (10.1186/s12885-018-4012-7) contains supplementary material, which is available to authorized users.

## Background

Glioblastoma (GBM; World Health Organization grade IV astrocytoma) is the most common malignant brain tumor with an annual incidence of 3.5 cases per 100,000 worldwide [1]. It is also one of the most lethal human cancers. The median overall survival is 12–15 months with standard treatment [2], and 3–6 months for patients with recurrent GBM [3]. Owing to uncertainty in clinical outcome in individual patients, new prognostic markers are needed for GBM patients, especially those with potential to affect patient outcome through druggable targets.

Endothelins are vasoactive peptides that exert their effects through interactions with the G-protein-coupled receptors endothelin receptor A (ETAR) and endothelin receptor B (ETBR). ETAR is expressed mainly in vascular smooth muscle cells and stromal cells, whereas ETBR is expressed mainly in endothelial cells; ETAR mediates vasoconstriction, and ETBR vasodilatation and also stimulates cell proliferation (reviewed in [4]. Dysregulation of ETBR has been implicated in cardiovascular disease and linked to a congenital disorder, Hirschsprung’s disease (reviewed in [4]). Moreover, ETBR is overexpressed in vulvar cancer [5], clear-cell renal cell carcinoma [6], and esophageal squamous cell carcinoma [7] and is closely associated with disease progression and poor patient survival [5–7]. Consistent with a crucial role for ETBR in tumorigenesis, some ETBR antagonists may be beneficial in treating melanoma or glioma [8–11].

Overexpression of ETBR in GBM was associated with a poor prognosis in a Chinese population [12]. Since ethnicity may play a major role in the pathogenesis of gliomas [13, 14], we investigated whether ETBR overexpression could be detected in patients with GBM and in other cancers outside of China, whether ETBR expression correlates with patient survival (and thus its potential use as a prognostic marker and/or therapeutic target), and whether clinically available endothelin receptor blockers/antagonists have toxic effects on cancer cells in vitro.

## Methods

### Patient cohort

Formalin-fixed, paraffin-embedded tissue sections were from 25 GBM cases, were selected from our previously studied cohort without prior selection [15]. Demographic information and clinical data with time to tumor progression (TTP) and overall survival (OS) for all GBM cases are shown in Table 1. Ten normal samples from aging control brains (frontal part of brain from men) median age 57 [50-61 yrs] were from the Department of Pathology, University of Malaya Medical Center (ethical number 896.7). The use of patient materials was approved by the Ethics Committee at the Karolinska Institutet and by the Medical Ethics Committee, University of Malaya Medical Center, Malaysia, and conducted in accordance with the Declaration of Helsinki.

**Table 1:** Demographic and available clinical information

Case number	TTP (months)	OS (months)	ETBR expression	Age (years)	Gender	Extent of resection	
						Radical	Partial
K7686-2004	4	5	3+	73	M	No	Yes
K9802-2004	5	5	1+	68	M	Yes	No
K4448-2004	1	14	1+	66	M	No	Yes
K12700-2004	1	5	1+	64	F	Yes	No
K10452-2004	7	10	1+	59	F	Yes	No
K5126-2004	3	7	1+	57	M	Yes	No
K11136-2004	12	20	1+	56	M	Yes	No
K17437-2004	16	17	2+	56	M	Yes	No
K4840-2004	7	20	1+	55	M	Yes	No
K16204-2004	10	11	1+	54	M	Yes	No
K9236-2004	12	15	1+	49	F	Yes	No
K16178-2004	12	13	1+	45	M	Yes	No
K3839-2004	12	14	3+	28	M	Yes	No
K16102-2004	48	48	1+	57	F	Yes	No
K17407-2004	48	48	1+	26	F	Yes	No
K10315-2004	52	52	1+	29	M	Yes	No
K3174-2004	15	19	3+	79	F	Yes	No
K16595/04	17	36	1+	53	F	Yes	No
K1716-2005	7	82	1+	43	M	Yes	No
K3349-2005	3	3	3+	79	F	No	Yes
K8622-2005	9	12	3+	59	F	Yes	No
K9731-2005	4	7	3+	52	F	Yes	No
K15725-2005	2	4	2+	54	M	Yes	No
K16886-2005	4.5	14.5	2+	38	F	No	Yes
K17972-2005	8.5	18	1+	63	F	Yes	No

*TTP* time to tumor progression, *OS* overall survival; Staining was graded as low (1+) or high (2+ and 3+)

### ETBR immunohistochemistry

Formalin-fixed, paraffin-embedded sections were analyzed by immunohistochemistry as described but with minor modifications [7]. In brief, the sections were deparaffinized and rehydrated in a graded series of ethanol, and antigen was retrieved with the Decloaking Chamber NxGen (Biocare Medical, Concord, CA, USA) and Antigen Retrieval Citra Plus solution (Biogenex, Emergo Europe, The Hague, The Netherlands) at 110 °C for 15 min. The sections were cooled to room temperature, equilibrated with Tris-buffered saline, pH 7.6, and subjected to a series of blocking steps with protein block (Dako Sweden, Stockholm, Sweden), Fc receptor blocker (Biogenex), and normal horse serum. The sections were then incubated with primary rabbit anti-ETBR (cat. no. E9905; 1:200, Sigma-Aldrich, Stockholm, Sweden) at 4 °C for 16 h, washed three times with Tris-buffered saline, and placed in 3% (*v*/v) H_2_O_2_ in water for 15 min at room temperature to quench endogenous peroxidase activity. After three washings with Tris-buffered saline, the sections were incubated with secondary anti-rabbit antibody conjugated to horseradish peroxidase (ImmPRESS kit, Vector Laboratories, Orton Southgate, Peterborough, UK). Immunoreactivity was revealed with diaminobenzidine (Innovex Biosciences, GENTAUR Europe BVBA, Belgium). The sections were then counterstained with hematoxylin, dried, and mounted with xylene-based mounting medium. Positive staining was graded as low or high as described [12].

### Analysis of data from the cancer genome atlas (TCGA) and the genome expression omnibus (GEO)

To evaluate the ETBR expression profile in GBM patients, we obtained primary and processed gene expression data for TCGA GBM cohort from The Broad Institute TCGA GDAC Firehose (https://gdac.broadinstitute.org/) using RTCGA (http://rtcga.github.io/RTCGA). Kaplan-Meier survival curves were plotted using the clinical information submitted for GBM patients in TCGA. GEO datasets related to GBM (GSE2223, GSE7696, GSE16011, GSE10878, GSE46016, GSE15824, GSE31262, GSE42656, and GSE50161) were analyzed for ETBR expression normalized to control. Among selected studies GSE2223 (origin: normal brain samples and glioblastoma), GSE7696 (origin: non-tumoral brain samples and glioblastoma samples with radiotherapy or TMZ/radiotherapy, age 27-70 years), GSE10878 (origin: normal tissue and primary glioblastoma tissue, age: 39-76 years), GSE46016 (origin: neural stem cells and human glioblastoma stem cells, age: 33-71 years), GSE15824 (origin: normal brain tissue & astrocytes and primary glioblastoma tissue, age: 35-70 years), GSE31262 (origin: non-tumoral brain tissue and primary glioblastoma tissue, age:33-71 years), GSE42656 (origin: adult control cerebellum and pediatrics glioblastoma, age:1-82 weeks), and GSE50161 (origin: normal brain samples and primary tumor (glioblastoma), age: 35-70 years) were analyzed. Other types of gliomas such as astrocytoma, oligodendroglioma, medulloblastoma were not considered for the analysis. The data was log2 transformed and then a differential analysis was performed using standard Limma function (R package for the analysis of differential gene expression [16]). The normalized data further scaled between 0 and 1 using formula Zi = xi - min(x) / max(x) - min(x), where x represent the expression.

### Protein–protein interaction network

Information on proteins that interact with ETBR was obtained with a protein neighborhood analysis tool [17]. The interacting partners were shown with Cytoscape, an online open source software tool to display molecular interaction networks and biological pathways [18]. Gene ontology of ETBR was obtained with a gene set enrichment tool and enrichment scores as described [19].

### Proof-of-concept: in vitro cytotoxic assays

To test whether endothelin receptor blockers affect GBM cell growth and toxicity in vitro, three drugs currently in use to treat pulmonary artery hypertension were used—ambrisentan (Letairis/Volibris), which selectively blocks the ETAR, and macitentan (Opsumit) and bosentan (Tracleer), which block both ETAR and ETBR—in standard viability assays with a CellTiter 96 Aqueous One Solution Cell Proliferation Assay system (Promega) as recommended by the manufacturer. We also tested BQ788, which is selective for ETBR, and ACT-132577, the active metabolite of macitentan. The ACT-132577, bosentan, BQ788 and macitentan used in this study were from Medchemexpress LCC (Princeton, NJ, USA), while ambrisentan was from Ark Pharm, Inc. (Libertyville, IL, USA). All the drugs were provided and checked by Medivir, Stockholm, Sweden. To test the effects of the drugs on cancer cells, we used primary GBM cells (GBM30, GBM42, GBM48, GBM392, and GBM398) and three GBM lines (U-251 MG, U-373 MG (Uppsala), and U-343 MGa). To test drug effects on normal cells, we used human umbilical vein endothelial cells (HUVECs, Lonza, CH-3930 Visp, Switzerland), MRC-5 lung fibroblasts (ATCC, LGC Standards, Middlesex, UK), and retinal pigment epithelial cells (a generous gift from Dr. Rich Stanton, Cardiff University). Since the endothelin axis (consisting of endothelins, ETAR, and ETBR) has also been implicated in breast cancer, we also tested the drugs on three breast cancer lines: MCF7, MDA-MA-231, and SK-BR-3. In brief, approximately 1 × 10^4^ cells/well were seeded onto 96-well plates and treated with twofold serial dilutions of drug (0.78–200 μM). Cell viability was assessed on day 6 with a VersaMax ELISA Microplate Reader (Molecular Devices, Wokingham, Berkshire, UK) at an optical density of 490 nm (reference wave length, 650 nm). The optical density of treated cells was expressed as a percentage of untreated cells, which were considered 100% viable.

### Statistical analysis

*P* < 0.05 was considered to indicate statistical significance. Survival curves were estimated with the Kaplan–Meier method, and the significance of differences between the curves was determined with the log-rank test. Boxplots were plotted with R programming and analyzed by *t* test.

## Results

### Expression data from TCGA and GEO database

**Fig. 1:**
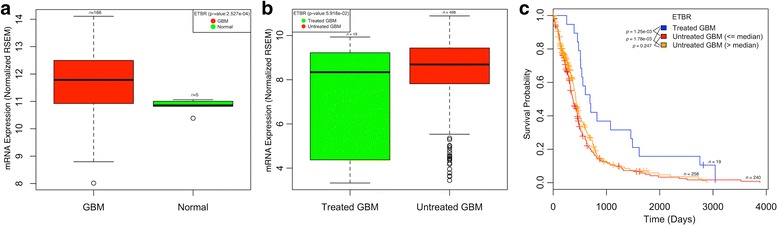
Endothelin receptor type B **(**ETBR) mRNA expression and its correlation with GBM patient’s survival as determined by bioinformatics analysis of the TCGA database. **a** ETBR mRNA expression was significantly higher in patients with GBM (*n = 166*) than in normal controls (*n = 5*) as normalized with RSEM (RNA-Seq by Expectation Maximization) software [35] **b** ETBR expression was higher in patients with untreated GBM than in those with treated GBM. **c** Survival curves based on clinical information and ETBR mRNA expression of treated and untreated GBM reported in the TCGA database

**Fig. 2:**
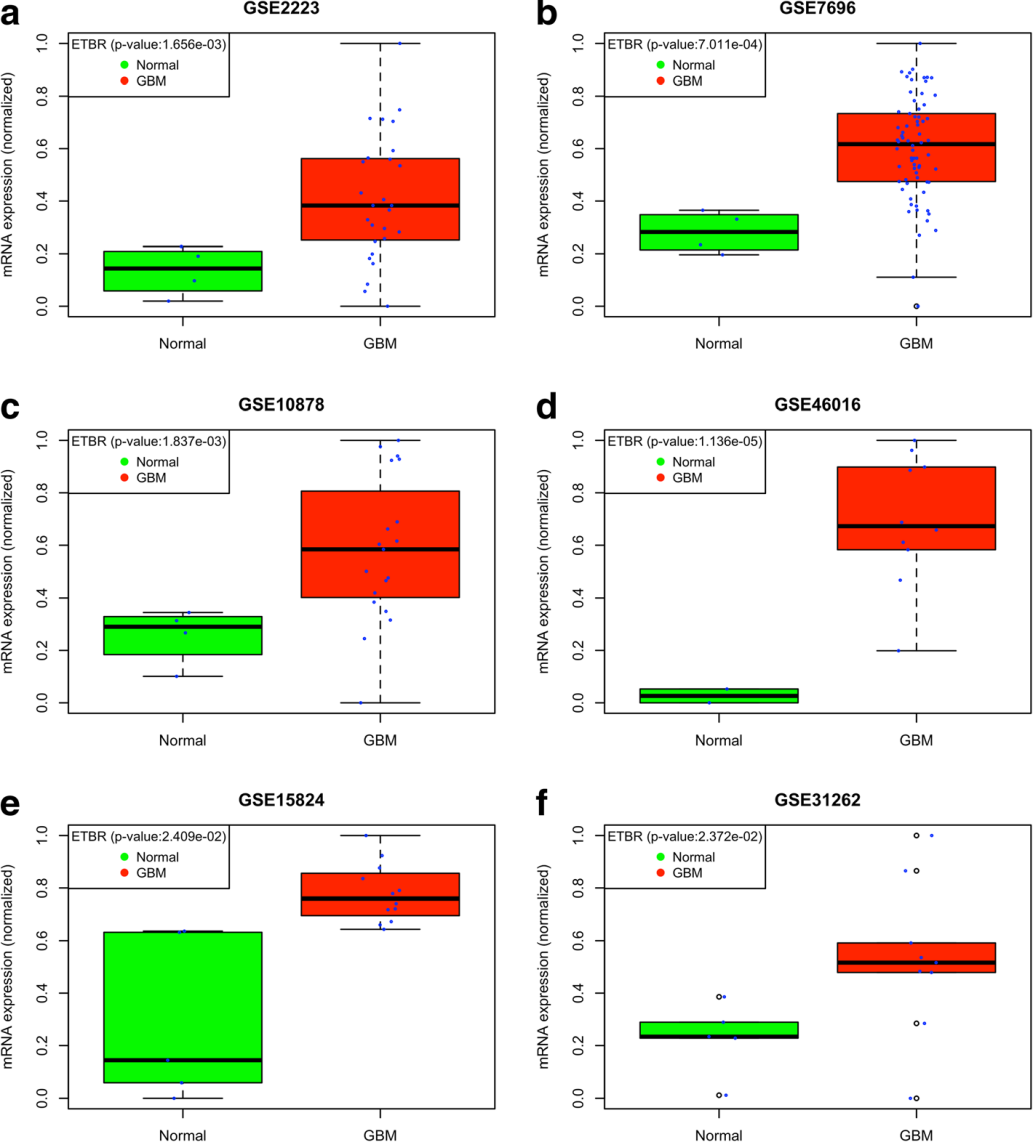
Normalized ETBR mRNA expression determined by analyzing GEO datasets (**a**-**f**). ETBR mRNA expression is shown for control (normal) and GBM patients. The signifance obtained from t-test is shown in inset box

To determine whether ETBR is overexpressed in GBM, we analyzed the ETBR mRNA expression in TCGA and GEO databases. In TCGA, mRNA expression data (*n* = 171) demonstrate that the median expression of ETBR was significantly higher in primary (de novo) GBM tumors than in normal aging control brains (Fig. 1a). Similarly, ETBR mRNA expression was higher in patients with untreated primary (de novo) GBM tumor than in tumors from patients treated for primary GBM (from the Affymetrix HuExGeneChip mRNA microarray data, quantile normalized; *n* = 517) (Fig. 1b). Interestingly, patients with untreated primary (de novo) GBM tumors with higher median expression of ETBR tended to have shorter survival compared to those with lower median expression (Fig. 1c). The survival data from GSE7696 and GSE16011 cohorts also showed that patients with over-median expression of ETBR had lower survival rates at 3 years than 5 years, respectively (Additional file 1: Figure S1). In the GEO datasets GSE2223, GSE7696, GSE10878, GSE46016, GSE15824, and GSE31262, ETBR expression was significantly higher in GBM patients than in controls (Fig. 2); no difference of ETBR expression was noted in datasets GSE42656, and GSE50161 (Additional file 1: Figure S2), perhaps mirroring the heterogeneity of the disease.

**Fig. 3:**
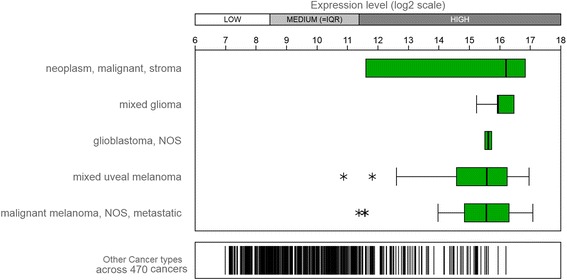
ETBR expression level in different cancers. The expression level of ETBR in human cancers is shown from Affymetrix Human Genome U133 Plus 2.0 Array. Enrichment of five major cancers in ETBR expression, shown with Genevestigator (http://genevestigator.com/gv/). NOS = Not Otherwise Specified, accordingly to WHO classification of CNS tumors (2016)

To further investigate the expression levels of ETBR in other cancers, we analyzed ETBR expression in silico with available datasets. ETBR expression varied among cancer types but was higher in malignant cancer, mixed glioma, GBM, and melanoma (Fig. 3 and Additional file 1: Figure S3). Taken together, these data predicted that overexpression of ETBR may be a prognostic marker for a subset of GBM patients and potentially for other tumor types as well (Additional file 1: Figure S3).

### ETBR is overexpressed in GBM

**Fig. 4:**
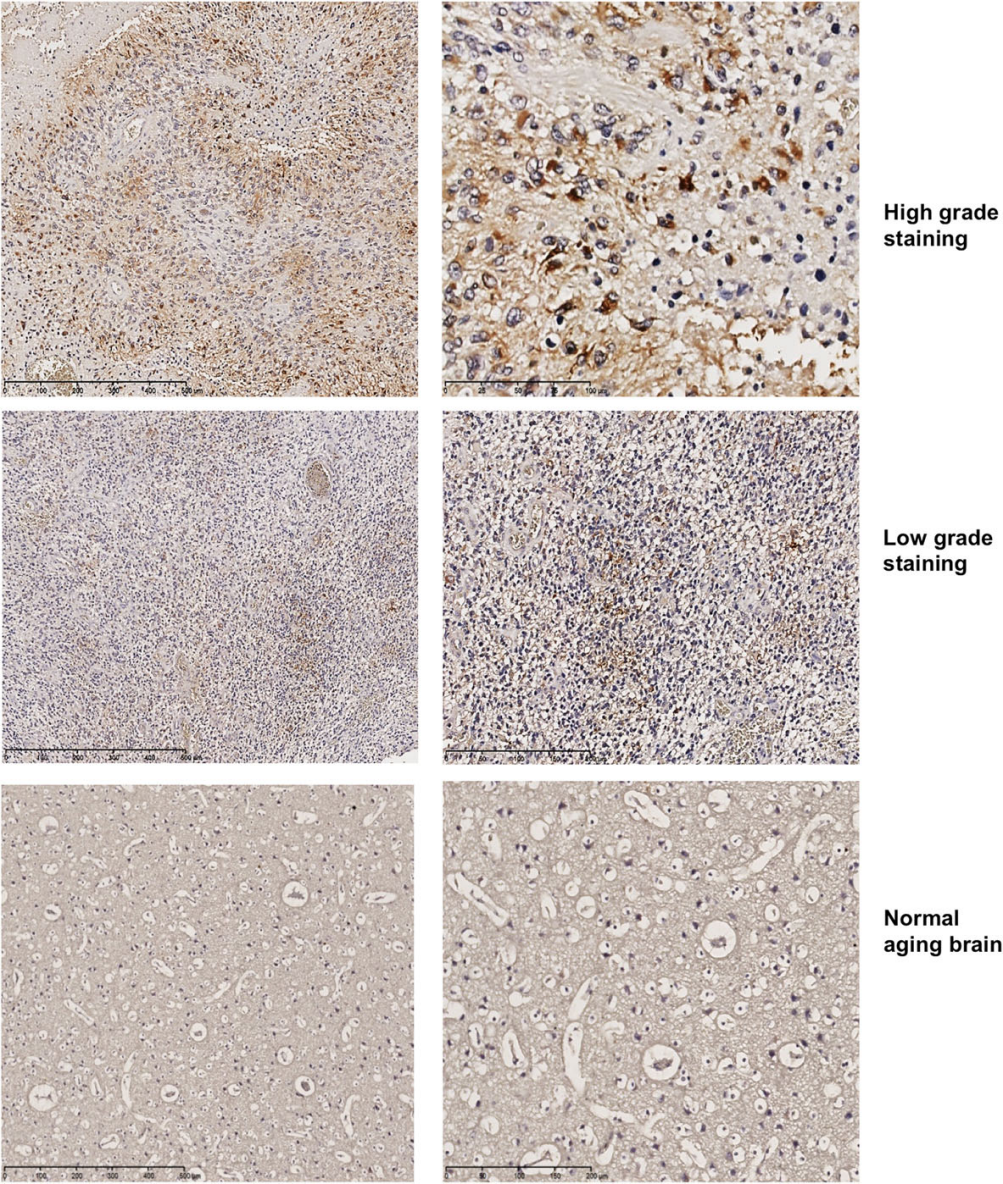
Protein expression of ETBR in GBM by immunohistochemistry staining. Representative photomicrographs showing high or low grade of ETBR staining (brown). Normal aging brains (frontal part) served as controls. Right panel is a higher magnification of the left panel

To confirm the higher expression of ETBR shown by the bioinformatics analysis in tissue specimens, we examined tumor tissue specimens obtained from 25 GBM patients we studied in a previous cohort [15] fro ETBR expression. ETBR expression was low in 64% (*n* = *16*) and high in 36% (*n* = *9*). ETBR was predominantly detected in the cytoplasm of tumor cells and was not found in adjacent nontumor cells, consistent with previous findings [20]. Little or no ETBR immunoreactivity was detected in control brains (*n* = *10*) (Fig. 4) and was mainly located in corpora amylacea and occasionally in some arteriole-like structures.

### Overexpression of ETBR is correlated with a shorter overall survival

**Fig. 5:**
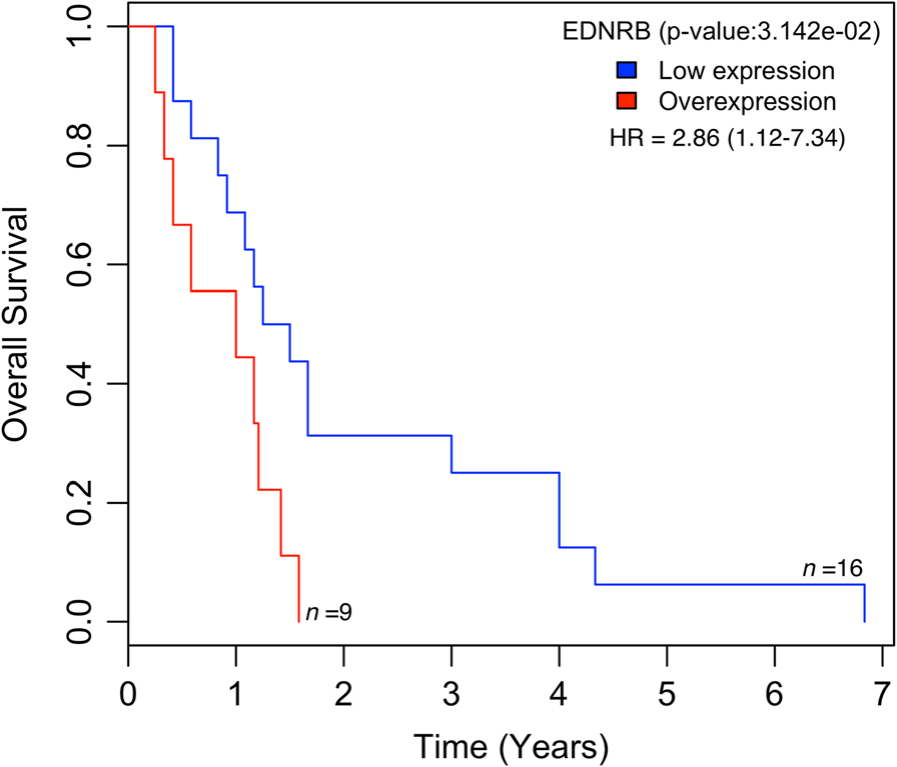
Kaplan-Meier survival curve for ETBR in 25 Swedish GBM patients. Overexpression of ETBR is correlated with shorter survival, whereas lower expression of ETBR is correlated with longer survival

To determine whether ETBR expression levels are of prognostic value for GBM patients, we used the Kaplan-Meier method to analyze the survival of 25 patients with low or high ETBR expression levels. ETBR expression correlated inversely and significantly with the survival times of these GBM patients (Fig. 5). Interestingly, we observed higher expression of ETBR in both ‘Classical’ and ‘Neural’ subtypes of GBM according to molecular classification [21] that tended to be correlated with poor overall survival (Additional file 1: Figure S5A-B).

### Druggability of ETBR

**Fig. 6:**
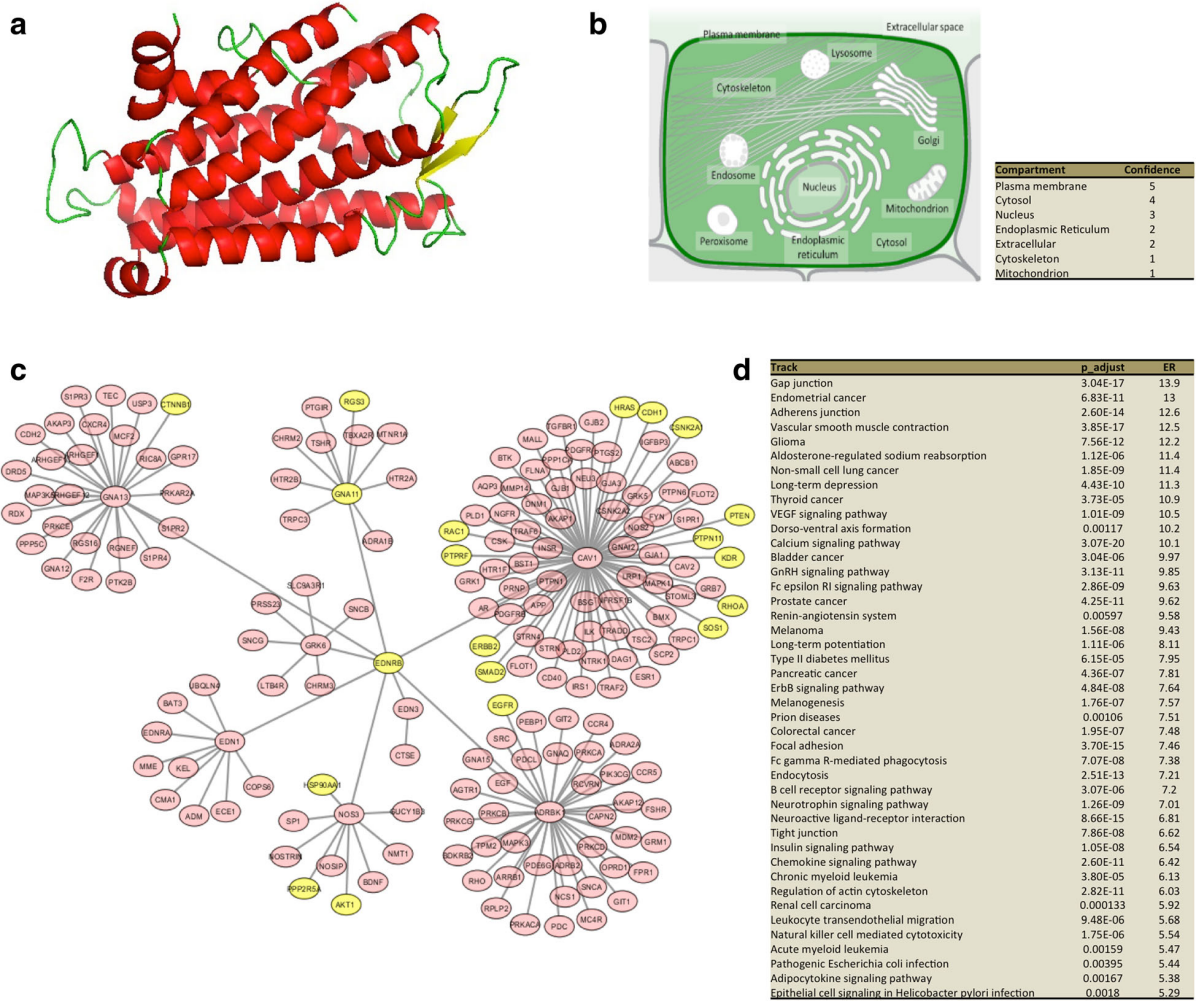
In silico analysis of the protein structure and cellular localization of ETBR protein and its protein–protein interactions. *A*) The SWISS model of ETBR protein (transmembrane, G protein-coupled receptor), residues 111–398. Helices are shown in red, sheets in yellow, and loops in green. *B*) ETBR is primarily localized to the plasma membrane, cytosol, and nuclear membrane as revealed by COMPARTMENTS resource (http://compartments.jensenlab.org). *C*) Analysis of protein–protein interactions shows that ETBR primarily interact with eight proteins, which interact with 175 proteins. *D*) Gene ontology of ETBR neighborhood proteins

Next, we used network neighborhood analysis to examine possible interaction networks and signaling pathways of ETBR. The general structure of ETBR is shown in Fig. 6a. ETBR is known to be expressed on the plasma membrane, in the cytosol and on the nuclear membrane (Fig. 6b). The analysis showed that ETBR potentially interacts with eight proteins: guanine nucleotide-binding protein subunit alpha-11, guanine nucleotide-binding protein subunit alpha-13, caveolin-1, G protein-coupled receptor kinase 6, endothelin-1, endothelin-3, adrenergic beta receptor kinase 1, and nitric oxide synthase 3 (Fig. 6c). Further expansion of protein neighbors showed 175 interacting partners, many of which are involved in cell-cell communications (gap junction, adherens junction), the vascular endothelial growth factor signaling pathway, and calcium signaling that is associated with cancer pathogenesis (Fig. 6d). In addition, the ETBR-interacting proteins (up to second neighbor) acted as signature genes in different cancers; 11 proteins were observed in melanoma, 10 proteins in lung and stomach adenocarcinoma, and 5 proteins in GBM (Additional file 1: Figure S4A) [22]. We also found an association between cancer types and ETBR-interacting proteins (Additional file 1: Figure S4B). Collectively, these data suggest that ETBR is a potential therapeutic target in GBM and other cancers.

### In vitro viability assay

**Fig. 7:**
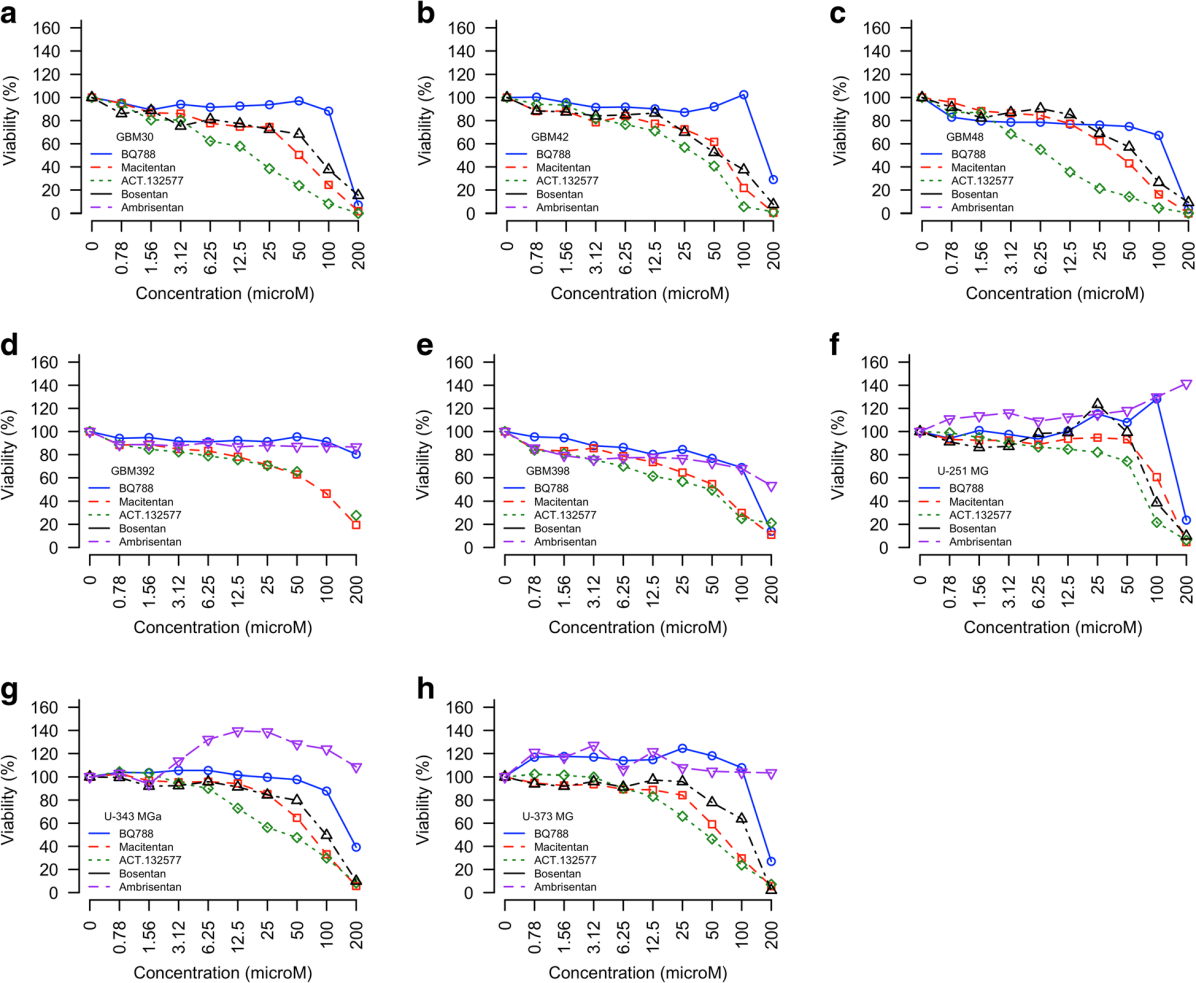
The effects of endothelin receptor antagonists on GBM. **a**–**e** Primary cells (*n = 3*). **f**–**h** Cell lines (*n = 6*)

**Fig. 8:**
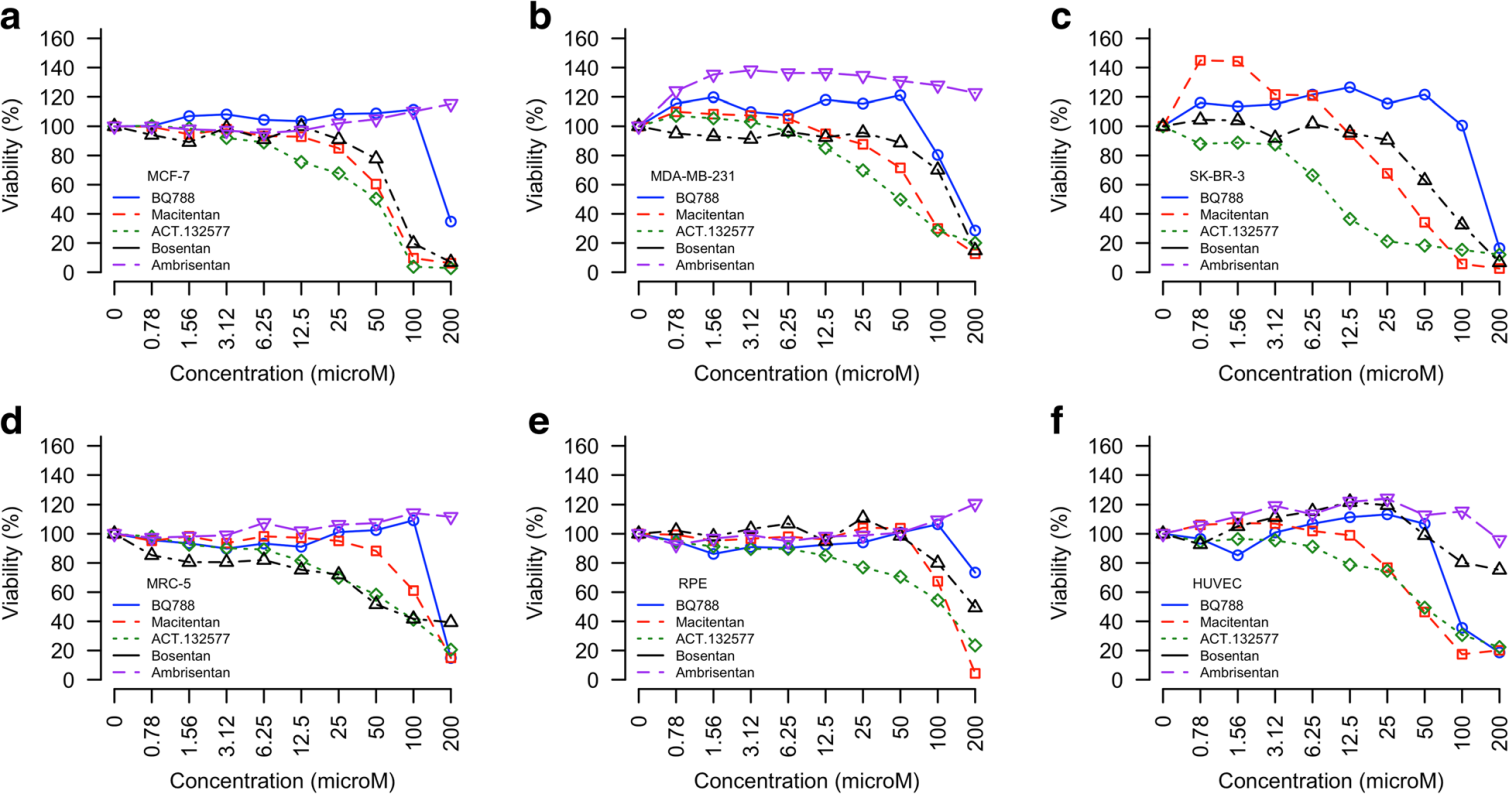
The effects of endothelin receptor antagonists on breast cancer cells and normal cells. **a**–**c** Breast cancer cell lines (*n = 6* for MCF-7 and MDA-MB-231; *n = 3* for SK-BR-3). **d–f**) Normal cells: MRC-5 fibroblasts (**d***, n = 6*), retinal pigment epithelial cells (RPE) (**e***, n = 6*), and human umbilical vein endothelial cells (HUVEC) (**f***, n = 6*)

To substantiate our prediction that ETBR is a druggable target for GBM and other cancer types, we used a standard viability assay to assess the cytotoxic effects of three clinically available endothelin receptor blockers—macitentan, bosentan, and ambrisentan—on primary GBM cells and GBM and breast cancer cell lines as compared with normal fibroblasts, endothelial and epithelial cells. Macitentan and its active metabolite (ACT-132577) reduced the viability of all GBM primary cells and cell lines tested; the effects were dose dependent (Fig. 7a–h). Bosentan had similar dose-dependent effects (Fig. 7a–c, f–h). In contrast, ambrisentan was not cytotoxic, even at the highest tested dose (Fig. 7d–h), apart from a minor trend toward reduced viability of GBM398 (Fig. 7e). Strikingly, at the highest dose, BQ788 dramatically reduced the viability of primary GBM392 cells and cell line U-343 MGa (Fig. 7a–h). Similar dose-dependent effects in breast cancer cells lines were observed for bosentan and for macitentan and its active metabolite ACT-132577; ambrisentan was not cytotoxic, and BQ788 had a single dramatic effect (Fig. 8a–c). Normal fibroblasts and epithelial cells tolerated ambrisentan and BQ788 well at 100 μM but not at 200 μM (Fig. 8d and e, respectively). Macitentan and its active metabolite resulted in 40–50% cell death at 100 μM in both cell types; HUVECs were more sensitive to macitentan and its metabolite (Fig. 8f). Notably, there is a differential baseline expression of ETBR in primary GBM tissues/cells and breast cancer lines compared to that of normal fibroblasts, endothelial and epithelial cells (Additional file 1: Figure S6). These findings suggest the potential feasibility of using endothelin receptor antagonists to treat GBM and breast cancer, and that fine-tuning of the ETAR and ETBR balance is crucial for maximum cytotoxicity while sparing normal cells.

## Discussion

In this study, we investigated whether ETBR is overexpressed in GBM tumors in a Swedish patient cohort and assessed the potential usefulness of ETBR as a prognostic marker and drug target for GBMs and other types of cancer. We found that ETBR is indeed often overexpressed in GBM tumors, with little or no immunoreactivity in control brains. Analysis of expression data from TCGA and a subset of GEO datasets showed that overexpression of ETBR in GBM was correlated with shorter patient survival. Similarly, by examining ETBR expression across 470 cancers, glioma or GBM were again found to have high expression. By mapping the protein neighborhood to ETBR, we found that ETBR is mainly predicted to interact with eight proteins that further interact with 175 additional proteins, many of which are involved in cell-cell communication (gap junction, adherens junction), the vascular endothelial growth factor signaling pathway, and calcium signaling—all of which are associated with cancer pathogenesis. These results support the potential use of ETBR blockers as a targeted therapy for cancer [10].

The endothelin axis has been implicated in the pathogenesis of many types of cancers (reviewed in [23]). In particular, ETBR is overexpresssed in bladder carcinoma [24], melanoma [25], small-cell lung cancer [26], vulvar cancer [5], clear-cell renal cell carcinoma [6], oesophageal squamous cell carcinoma [7], and astrocytoma (including GBM) [12]. ETBR was also earlier reported to be highly expressed in melanoma [25]. Of note, ETBR overexpression was correlated with shorter patient survival or poor patient outcome in small-cell lung cancer, vulvar cancer, clear-cell renal cell carcinoma, esophageal squamous cell carcinoma, and GBM [5–7, 12, 24, 27] and may thereby represent a potential prognostic marker as well as a therapeutic target for several cancer forms. We confirmed this hypothesis in the current study. We assessed the toxicity of ETBR and ETAR blockers for cancer cells of different origins. While Ambrisentan was not cytotoxic to GBM cells or breast cancer cells, the ETBR-selective blocker BQ788, the dual ETBR and ETAR blockers bosentan and macitentan, and the active metabolite of macitentan, ACT-132577 inhibited tumor cell growth to some extent. The affinity of ambrisentan to ETBR is at most 1% of its affinity to ETAR (IC_50_ = 1 nM) (reviewed in [28]), and ambrisentan was the least effective of the drugs we tested. We speculate that the ratio between ETAR and ETBR may be crucial for maximum cytotoxic efficiency.

To our knowledge, only two studies have earlier demonstrated overexpression of ETBR in GBM tumors, one study of Han-Chinese patients [12] and one in Japanese patients [29]. Ethnicity is a factor in the pathogenesis of gliomas [13, 14]. Epidemiological data suggest that the incidence of glioma in the United States is higher among whites, followed by blacks, Hawaiians, Chinese or Japanese, Filipinos, and Alaskan natives [30]. The incidence of gliomas is also higher in Scandinavian countries than in Asian countries [30]. The ethnicity difference may be related to or result in alterations in the expression of key proteins. The promoter methylation status of the *O*^*6*^-methylguanine methyltransferase, a key DNA repair enzyme predicted the outcome of treatments that include an alkylating agent in Caucasian populations but not in Indians [31]. In the present study, we detected higher ETBR immunoreactivity in tumor cells from GBMs of Swedish patients, while little or no ETBR immunoreactivity was detected in adjacent nontumor cells, which consistent with a previous report [20]. ETBR is known to predominantly express by astrocytes, where it helps regulate cell hypertrophy [32]. In normal aging brains, we detected ETBR immunoreactivity in corpora amylacea, which are glycoproteinaceous inclusion bodies associated with aging or neurodegenerative diseases. The significance of this finding is unknown but it is well-known that the corpora amylacea is immunoreactive to various proteins (reviewed in [33]) and recently, to an antibody against a late antigen (MAB8127, Millipore) of human cytomegalovirus, a ubiquitous beta herpesvirus [34].

Our study is limited by the relatively small number of patients. Hence a large-scale patient cohort is needed to further evaluate the usefulness of ETBR as prognostic marker. A future study should also be tailored to better understand the role of ETBR in the pathogenesis of GBM. Nevertheless, our study confirms that ETBR is overexpressed in GBM and other cancer forms and further implicates ETBR as a potentially useful prognostic marker and possibly a therapeutic target for cancer.

## Conclusion

This study examined the potential role of ETBR in GBM tumors as well as in other cancer forms. ETBR expression was higher in GBM tumors and several other cancer forms than in control tissues and high ETBR expression was correlated with poor patient outcome. ETBR blockers were in general more toxic to tumor cells than normal cells, which imply a potential benefit of ETBR blockers in cancer therapy.

## Additional file

Additional file 1: Figure S1. Survival curves based on clinical information obtained from GSE7696 and GSE16011 for Endothelin receptor type B (ETBR) mRNA expression in GBM. GSE7696 and GSE16011 cohort shows that over-median expression of ETBR has lower survival rate at 3 years than 5 years (http://watson.compbio.iupui.edu/). **Figure S2.** ETBR mRNA expression in GSE42656 (A) and GSE50161 (B). No statistically significant difference was observed for ETBR expression in normal brain tissue compared to that of GBM patients, mirroring heterogeneity of disease. **Figure S3.** Overexpression of ETBR mRNA in different cancers of the TCGA cancer cohort is shown using The Cancer Cell Line Encyclopedia (CCLE, https://portals.broadinstitute.org/ccle). Numbers in parentheses indicate sample size. **Figure S4.** The ETBR-interacting proteins (up to second neighbor) were searched in different cancer signature genes as described [22] *A*). The number of ETBR-interacting proteins found to be signature genes in various cancer cohorts were shown. *B*) The association between cancer types and ETBR-interacting proteins suggests a possible role of ETBR in GBM and other cancers. Cancer types are shown with degree-based node shape and surrounding red circles, whereas ETBR-interacting proteins are shown with degree based nodes (orange). **Figure S5.** ETBR expression in different subtypes of GBM (A) and its correlation with survival (B) according to molecular classification [21]. Higher expression of ETBR in both ‘Classical’ and ‘Neural’ subtype tended to be correlated with poor overall survival. **Figure S6.** Relative expression of ETBR in various primary GBM cells, breast cancer lines (MCF-7, SKBR3 and MDA-MB-231), fibroblast (MRC-5), endothelial cells (HUVEC) and epithelial cells (RPE) as normalized to MRC-5. (DOC 2199 kb)

## Abbreviations

ERA: Endothelin receptor antagonists
ETAR: Endothelin A receptor
ETBR: Endothelin B receptor
GBM: Glioblastoma
