# Supplementary material for: Overexpression of endothelin B receptor in glioblastoma: a prognostic marker and therapeutic target?
Source: BMC Cancer. 2018 Feb 6;18:154. doi: 10.1186/s12885-018-4012-7 (PMC5801893; doi:10.1186/s12885-018-4012-7)
Supplement: Additional file 1: Figure S1. — Survival curves based on clinical information obtained from GSE7696 and GSE16011 for Endothelin receptor type B (ETBR) mRNA expression in GBM. GSE7696 and GSE16011 cohort shows that over-median expression of ETBR has lower survival rate at 3 years than 5 years (http://watson.compbio.iupui.edu/). Figure S2. ETBR mRNA expression in GSE42656 (A) and GSE50161 (B). No statistically significant difference was observed for ETBR expression in normal brain tissue compared to that of GBM patients, mirroring heterogeneity of disease. Figure S3. Overexpression of ETBR mRNA in different cancers of the TCGA cancer cohort is shown using The Cancer Cell Line Encyclopedia (CCLE, https://portals.broadinstitute.org/ccle). Numbers in parentheses indicate sample size. Figure S4. The ETBR-interacting proteins (up to second neighbor) were searched in different cancer signature genes as described [22] A). The number of ETBR-interacting proteins found to be signature genes in various cancer cohorts were shown. B) The association between cancer types and ETBR-interacting proteins suggests a possible role of ETBR in GBM and other cancers. Cancer types are shown with degree-based node shape and surrounding red circles, whereas ETBR-interacting proteins are shown with degree based nodes (orange). Figure S5. ETBR expression in different subtypes of GBM (A) and its correlation with survival (B) according to molecular classification [21]. Higher expression of ETBR in both ‘Classical’ and ‘Neural’ subtype tended to be correlated with poor overall survival. Figure S6. Relative expression of ETBR in various primary GBM cells, breast cancer lines (MCF-7, SKBR3 and MDA-MB-231), fibroblast (MRC-5), endothelial cells (HUVEC) and epithelial cells (RPE) as normalized to MRC-5. (DOC 2199 kb) [file 12885_2018_4012_MOESM1_ESM.doc]

**Supplementary Information**


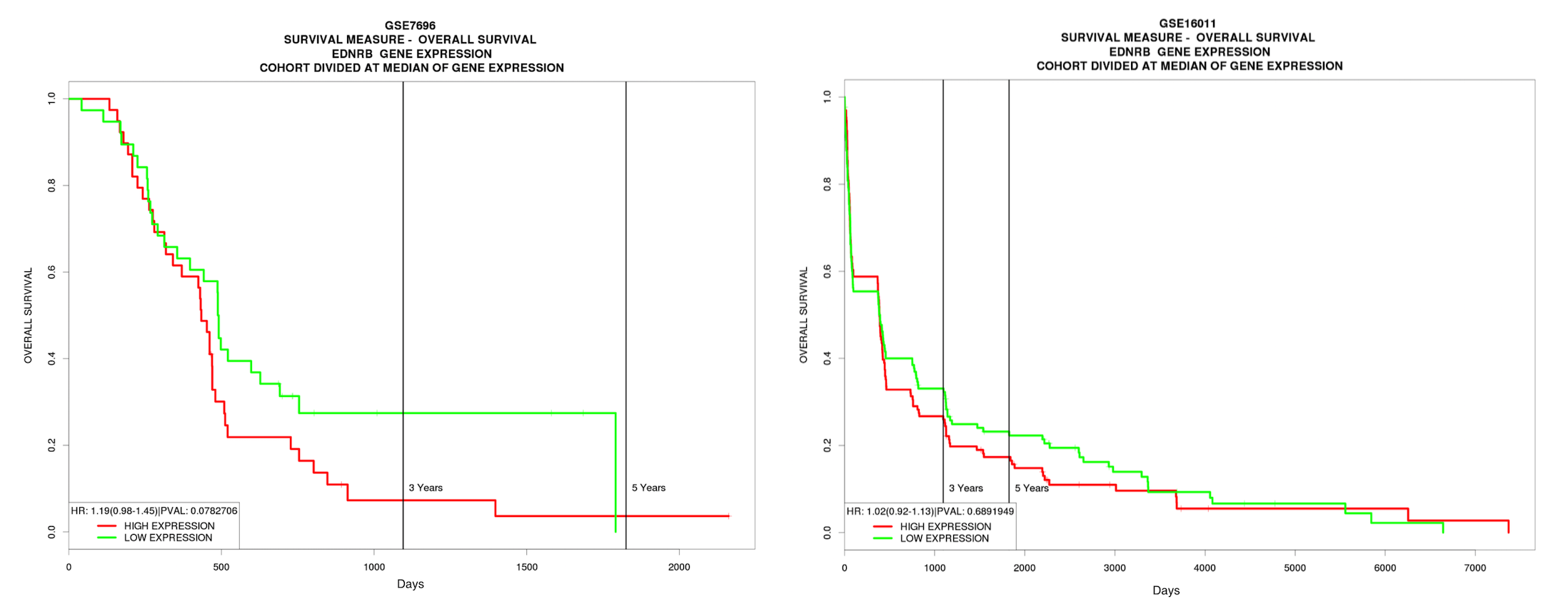


**Figure S1.** Survival curves based on clinical information obtained from GSE7696 and GSE16011 for Endothelin receptor type B (ETBR) mRNA expression in GBM. GSE7696 and GSE16011 cohort shows that over-median expression of ETBR has lower survival rate at 3 years than 5 years (<http://watson.compbio.iupui.edu/>).


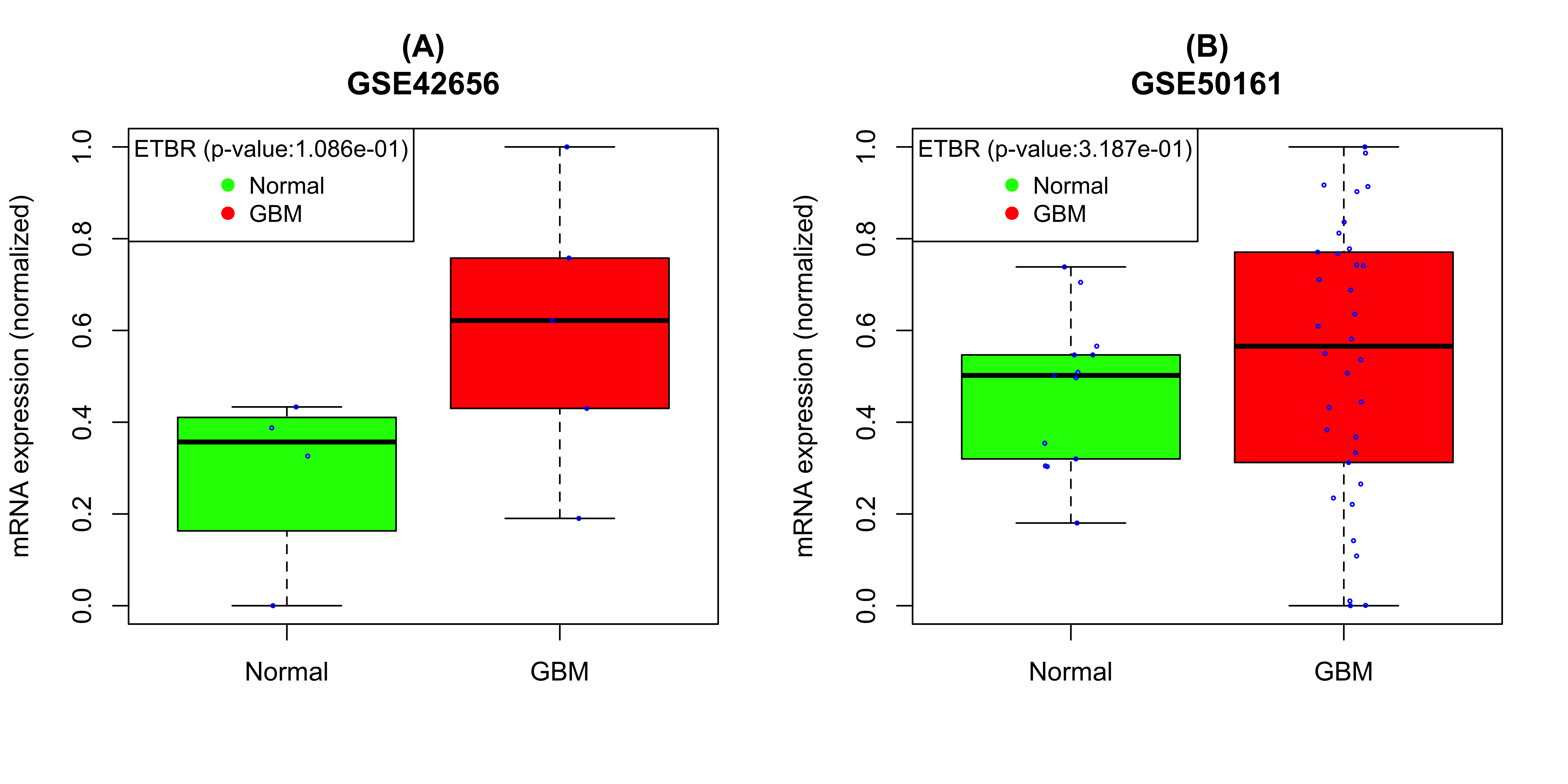


**Figure S2.** ETBR mRNA expression in GSE42656 (A) and GSE50161 (B). No statistically significant difference was observed for ETBR expression in normal brain tissue compared to that of GBM patients, mirroring heterogeneity of disease.


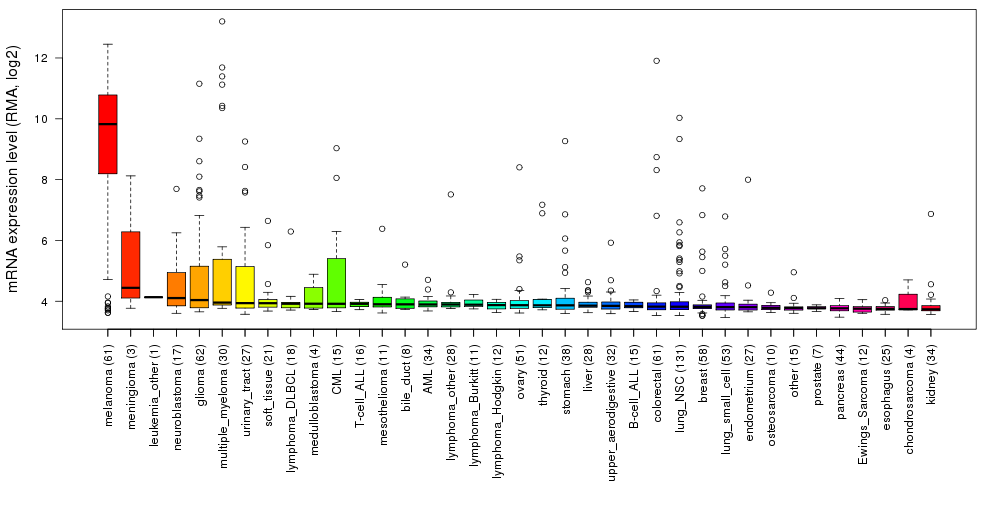


**Figure S3.** Overexpression of ETBR mRNA in different cancers of the TCGA cancer cohort is shown using The Cancer Cell Line Encyclopedia (CCLE, <https://portals.broadinstitute.org/ccle>). Numbers in parentheses indicate sample size.


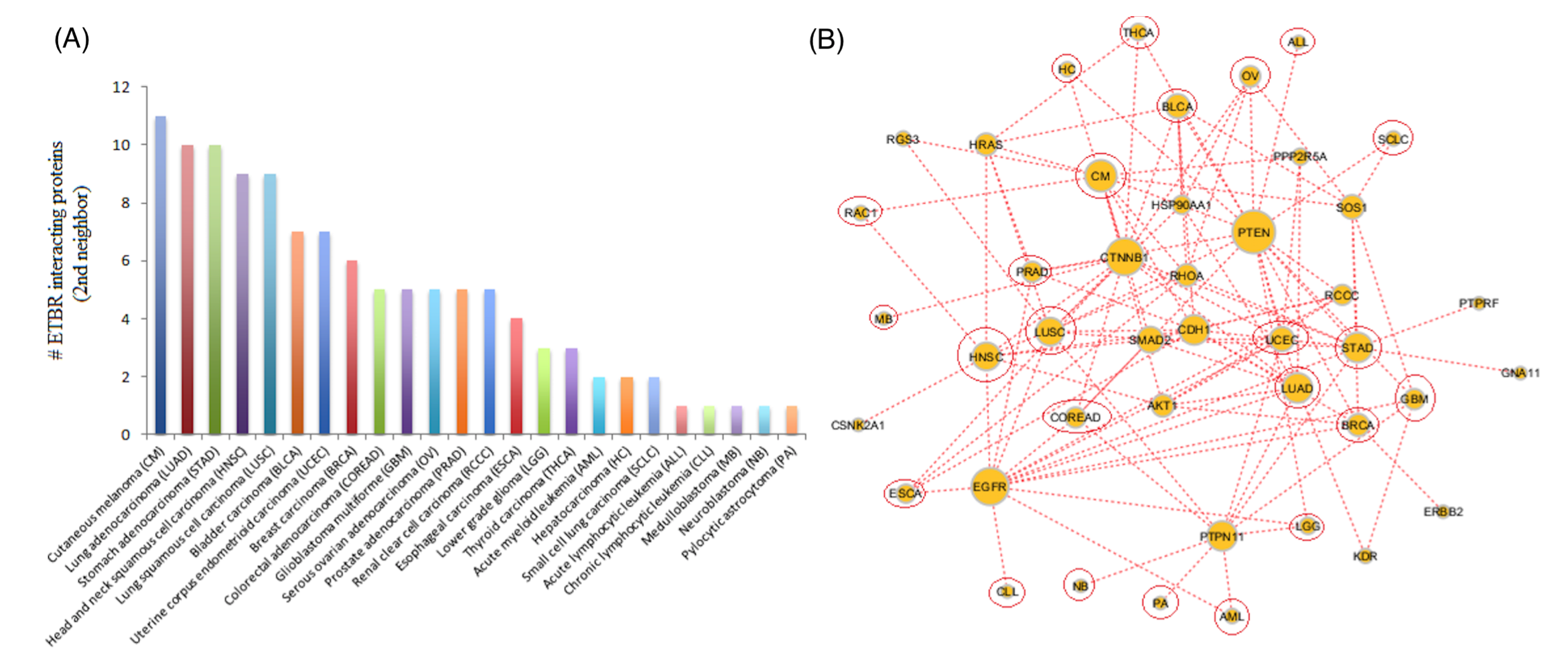


**Figure S4.** The ETBR-interacting proteins (up to second neighbor) were searched in different cancer signature genes as described *A*). The number of ETBR-interacting proteins found to be signature genes in various cancer cohorts were shown. *B*) The association between cancer types and ETBR-interacting proteins suggests a possible role of ETBR in GBM and other cancers. Cancer types are shown with degree-based node shape and surrounding red circles, whereas ETBR-interacting proteins are shown with degree based nodes (orange).


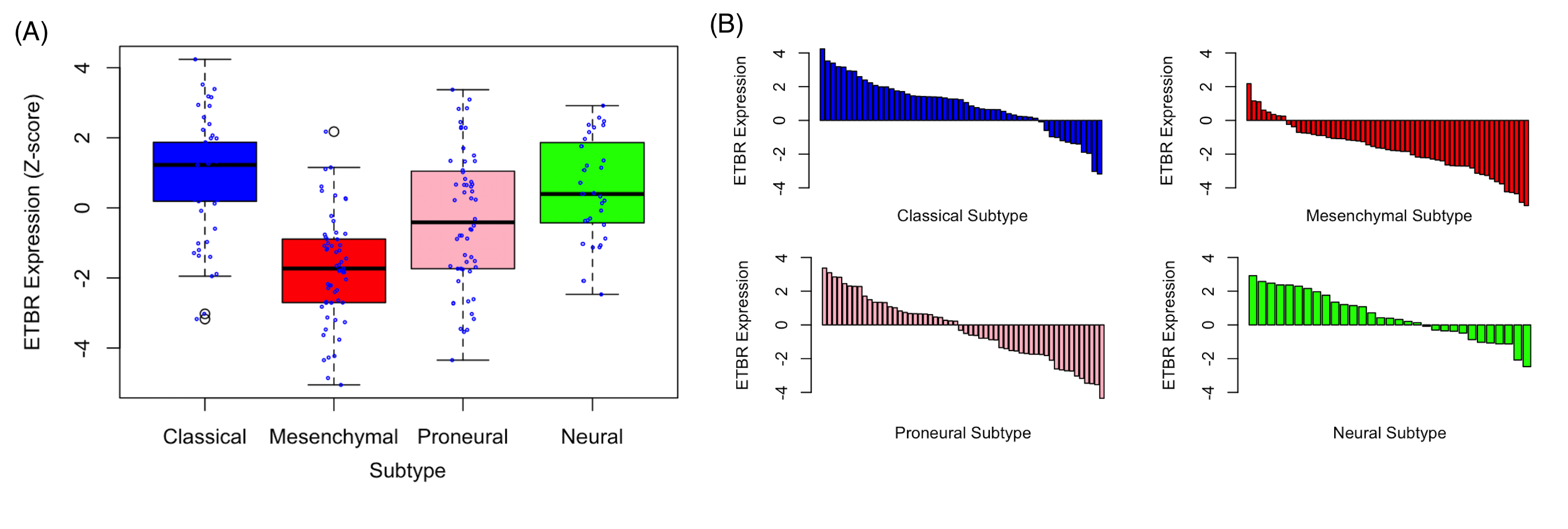


**Figure S5.** ETBR expression in different subtypes of GBM (A) and its correlation with survival (B) according to molecular classification . Higher expression of ETBR in both ‘Classical’ and ‘Neural’ subtype tended to be correlated with poor overall survival.


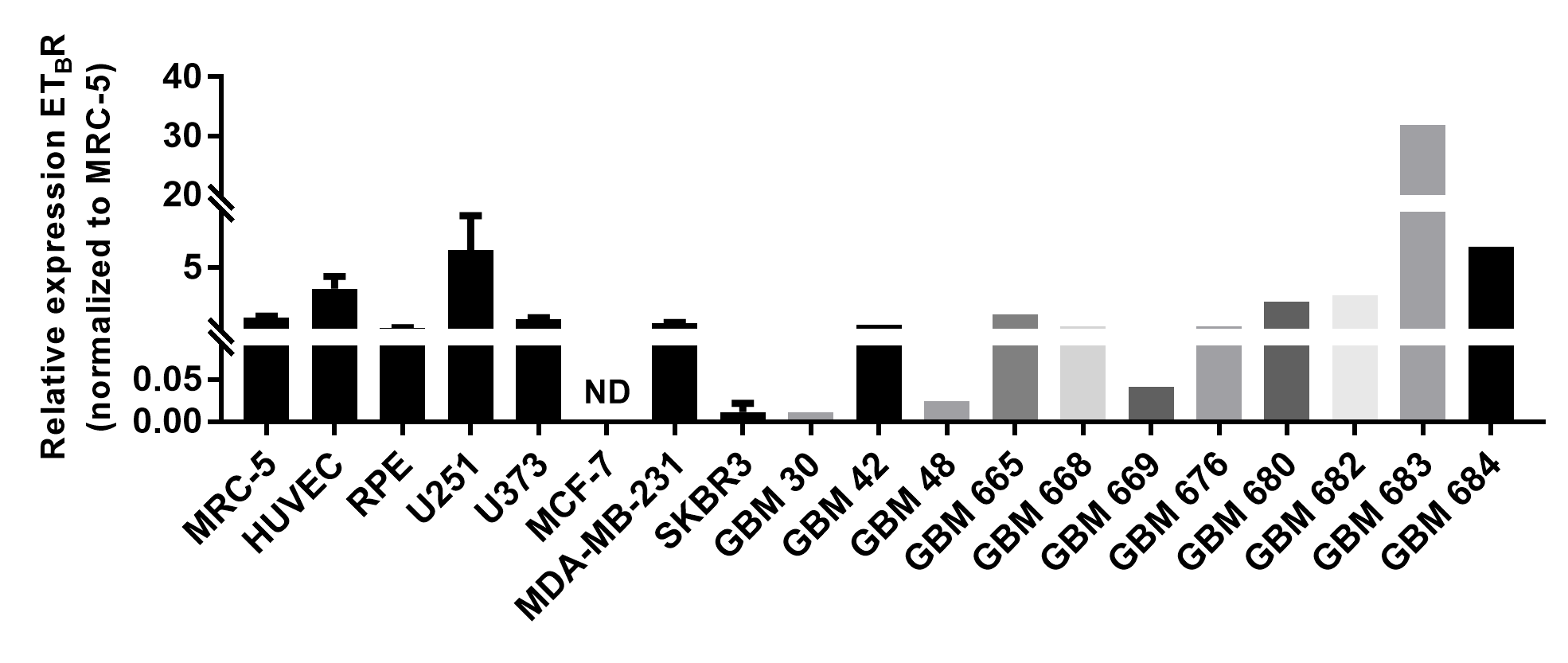


**Figure S6.** Relative expression of ETBR in various primary GBM cells, breast cancer lines (MCF-7, SKBR3 and MDA-MB-231), fibroblast (MRC-5), endothelial cells (HUVEC) and epithelial cells (RPE) as normalized to MRC-5.
